# Simple molecular dynamics simulation of hydrogen adsorption on ZSM 5, graphite nanofiber, graphene oxide framework, and reduced graphene oxide

**DOI:** 10.1016/j.heliyon.2021.e08528

**Published:** 2021-12-02

**Authors:** Jaka Fajar Fatriansyah, Donanta Dhaneswara, Iping Suhariadi, Muhammad Ihsan Widyantoro, Billy Adhitya Ramadhan, Muhammad Zaky Rahmatullah, Rahman Hadi

**Affiliations:** a Department of Metallurgical and Materials Engineering, Faculty of Engineering, Universitas Indonesia, Jawa Barat, 16424, Depok, Indonesia; b Department of Industrial Engineering, Faculty of Engineering, Bina Nusantara University, 11480, Jakarta, Indonesia

**Keywords:** Hydrogen uptake, Molecular dynamics, Largescale atomic/molecular massively parallel simulator, ZSM5, Graphite nanofiber, Graphene oxide framework, Reduced graphene oxide

## Abstract

The search for the most efficient materials that can store hydrogen has been challenged by various impediments in experimental studies, such as cost and complexity. Simulation study offers an easy method to overcome this challenge, but primarily requires powerful computing resources. In this paper, a simple MD simulation using a Large-scale Atomic/Molecular Massively Parallel Simulator (LAMMPS) was developed to calculate the hydrogen uptake in ZSM5, Graphite Nanofiber, Graphene Oxide Framework, and reduced Graphene Oxide. The method offered a more affordable computational method and relatively straightforward approaches while maintaining a high degree of accuracy and efficiency. The comparisons between simulation and experimental results were also presented. Based on our simulation, the calculations generally agreed with the results of experiments conducted for all the materials.

## 1 Introduction

The increase in high energy consumption is one of the most pressing global issues today, exacerbated by a decline in conventional energy production. The world needs alternative renewable energy resources that have minimal effects on climate change and global warming, unlike that of conventional energy sources. One of such proposed alternative energy resources is hydrogen, which has the highest gravimetric energy density, and can be used in a fuel cell to produce electricity and power vehicles. However, the imminent commercialization of hydrogen fuel cell vehicles does face challenges, since none of the hydrogen storage systems fully meet all the industry requirements (Arifin et al., 2021). To date, there still remain several concerns about hydrogen storage media such as media safety, and the undeveloped infrastructure of production, distribution, and charging equipment (Lin et al., 2012). Therefore, further research in hydrogen storage media is necessary to find the most effective and safest material capable of storing hydrogen (Thomas, 2007).

Physical adsorption has been proposed to be the most promising storage method so far, capable of upholding and releasing hydrogen (Patchkovskii et al., 2005). The method utilizes various solid materials, especially carbonaceous materials (Nagar et al., 2017). Of these, nanoporous materials such as carbon-based nanomaterials (CBN) and Zeolite have been reported to be the most favored due to their high adsorption surface area (Xia et al., 2013).

Molecular dynamics (MD) is a valuable tool used to study the interaction between hydrogen and the storage materials. In fact, it is one of the widely used atomic simulation tools with the advantage of determining characteristics of the receptor site by coding its chemical structure after optimizing the fundamental energy of the systems (Bouazizetal, 2019; Tran and Wang, 2017). However, this MD simulation is expensive, as it requires a high-performance computer and several steps. MD using Largescale Atomic/Molecular Massively Parallel Simulator (LAMMPS) offers a more affordable computational method to run a billion atom systems in a single workstation with a more straightforward approach, while maintaining a high degree of accuracy and efficiency (Agarwal et al., 2013).

Despite the simplicity and effectiveness of LAMMPS, only a few studies have been carried out so far on hydrogen adsorption behavior in CBN and Zeolite using this method. All these studies focus on the effects of nanostructured morphology on hydrogen storage efficiency and the impact of adsorbed hydrogen atoms on the crack-like structural defects (Jiang et al., 2015a, 2015b; Tabarraei et al., 2016; Zhu and Li, 2014). In experimental research, the commonly used methods to measure hydrogen adsorption are gravimetric and volumetric; the former method being where the system determines the amount of material to be dosed by weight, and the latter being where the system doses material according to the space it occupies (Keller and Staudt, 2005). To our knowledge, there has not been a computational study on the effects of pressure and temperature on the hydrogen adsorption characteristics of CBN and Zeolite using LAMMPS-based MD. In this study, we investigate the hydrogen adsorption characteristics in ZSM-5, graphite nanofibers (GNF), graphene oxide framework-32 (GOF-32), and reduced graphene oxide (rGO), representing a range of CBN materials under various pressures and temperatures, using LAMMPS-based MD.

## 2 Materials and methods

The simulation was conducted in LAMMPS Molecular Dynamics Simulator, available for download online (LAMMPS Molecular Dynamics Simulator, 2020). The materials were built using Avogadro (2020) with a feature termed the Super Cell Builder. ZSM-5 was built in Avogadro using a database from IZA (IZA Structure Commission, 2020). GNF was created directly from Avogadro, while GOF and rGO were created from Graphene Oxide Generator and Python-27, respectively.

The adsorbent materials and hydrogen molecules were combined in one system using Notepad++ to be processed in the LAMMPS. Lennard-Jones (LJ) interaction was used with a cut-off value of 4.0 in the LJ unit. The pair coefficient of the materials was obtained from previous studies (Mashayak and Aluru, 2012; Banerjee, 2008). The NVE setting was used with a time-step of 0.0003, which is equal to 6.468 × 10^−15^ s (one time-step is equal to 2.156 ps). All the simulations were conducted at the isothermal condition. The simulation temperature and pressure varied according to the material. The number of hydrogen molecules in the simulation was calculated using the Van der Waals equation, as follows:

(1) $$\left(P+\frac{n^{2}a}{v^{2}}\right)\left(V-nb\right)=nRT$$

where *P* is pressure, n is mole number, *a* and *b* are Van der Waals coefficients (*a* = 2.476 × 10^*−*2^ m ^6^Pamol^*−*2^ and *b* = 2.661 × 10^*−*5^m^3^mol^*−*1^), *V* is the volume, *R* is the gas constant (8.314 J/mol.K), and *T* is the temperature. The number of hydrogen molecules was continuous variables, and was generated by the simulator program using Eq. (1). The size of the simulation box (domain) was 2.16 × 10^−25^ m^3^. The code was run at certain time-steps until the system reached the equilibrium state, i.e., the number of hydrogen molecules inside the materials was averagely constant. The pressure dependent hydrogen uptake was calculated using the ratio between the mass of hydrogen molecules adsorbed/inside the materials, and the total mass of the materials.

## 3 Results and discussion

**Figure 1:**
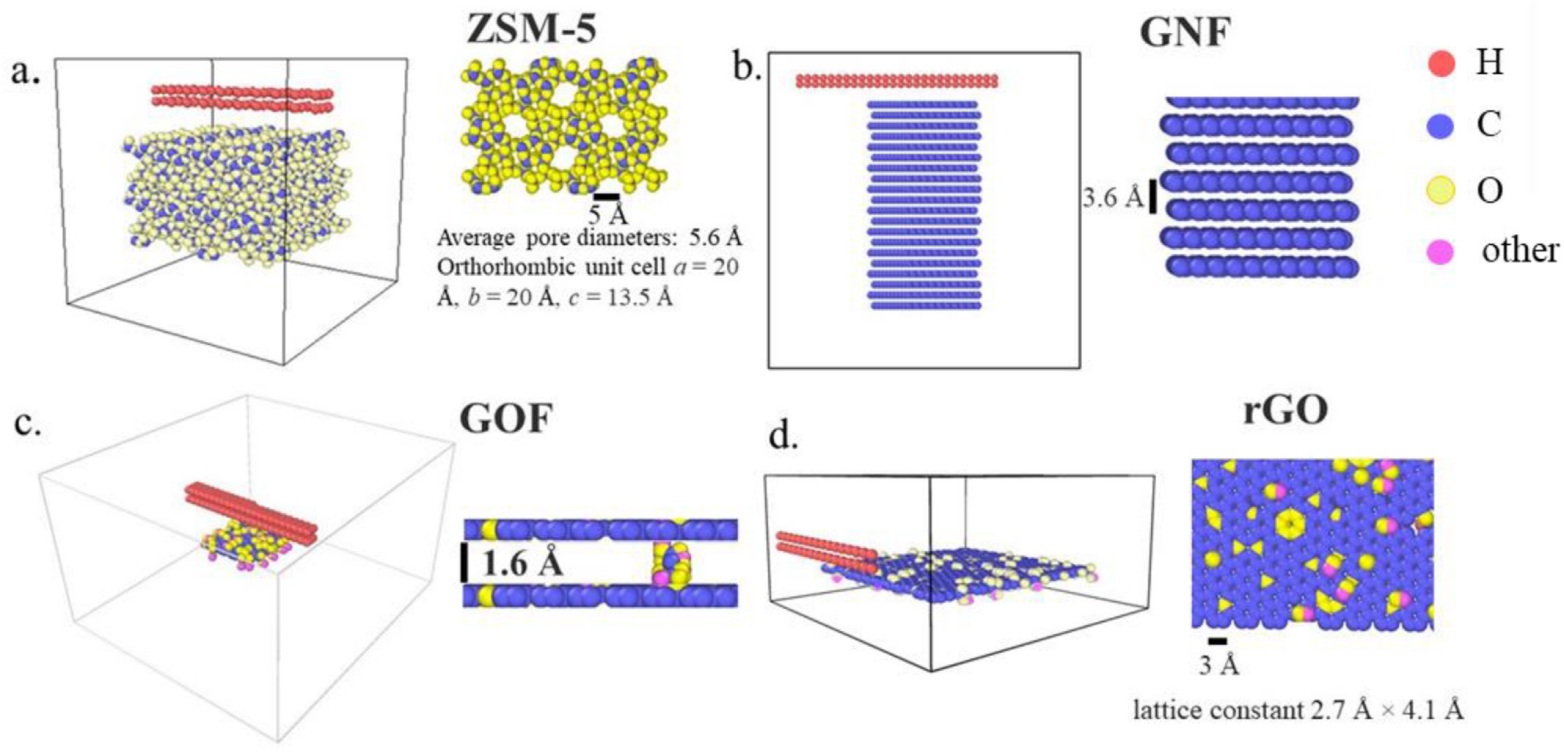
The visualization of the simulation boxes in the initial state for (a) ZSM-5, (b) GNF, (c) GOF-32, and (d) rGO.

**Figure 2:**
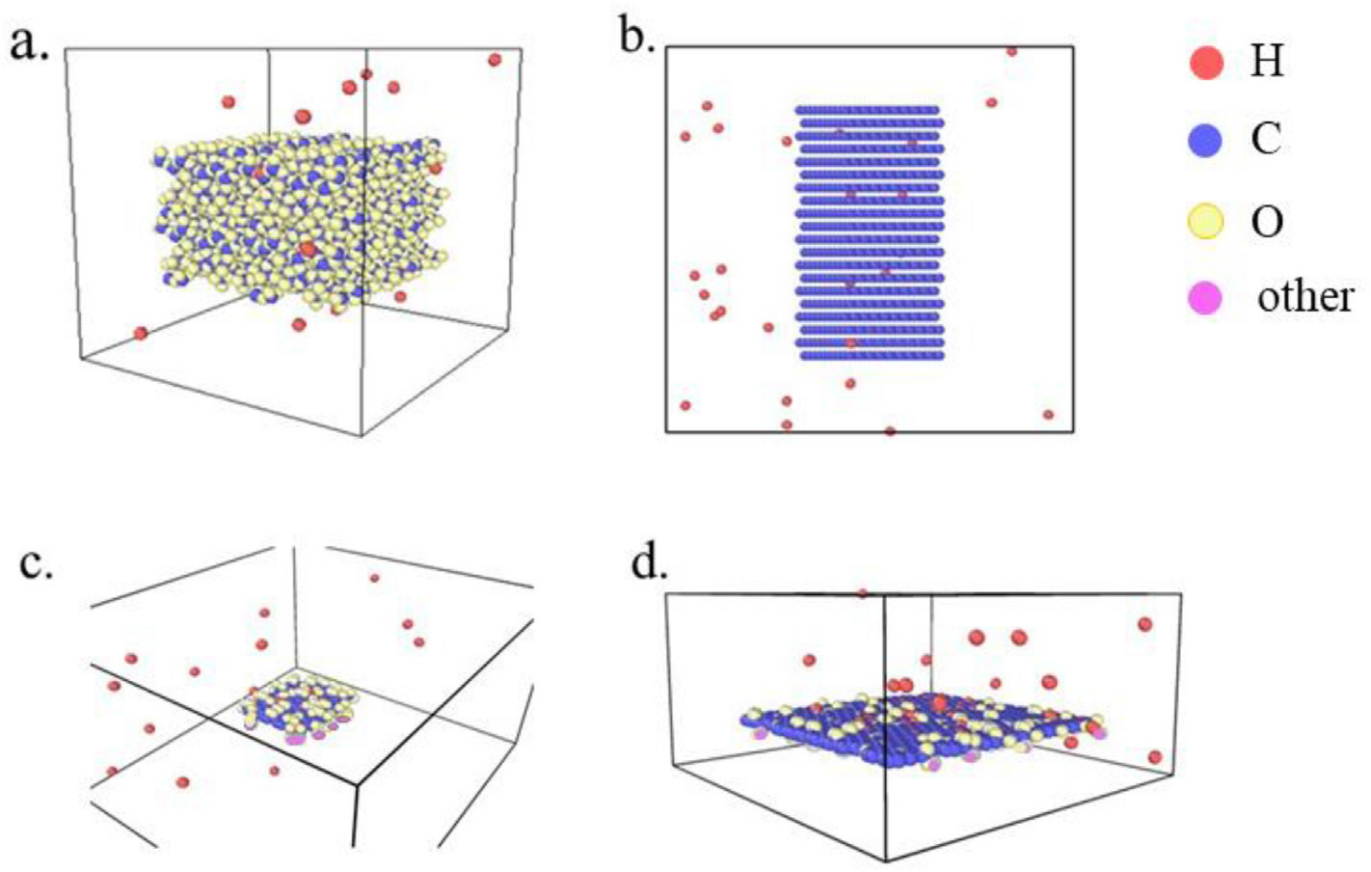
The visualization of the simulation boxes in the final state for (a) ZSM-5, (b) GNF, (c) GOF-32, and (d) rGO.

Figure 1a, b, c, and d show the visualizations of the simulation boxes and material structures for ZSM-5, GNF, GOF, and rGO in their initial states, respectively. We observe from the diagram that in the initial state, the hydrogen atoms are separated from the adsorbents. The simulations were run until the equilibrium state was reached. The final states of the simulations are shown in Figure 2a, b, c, and d.

**Figure 3:**
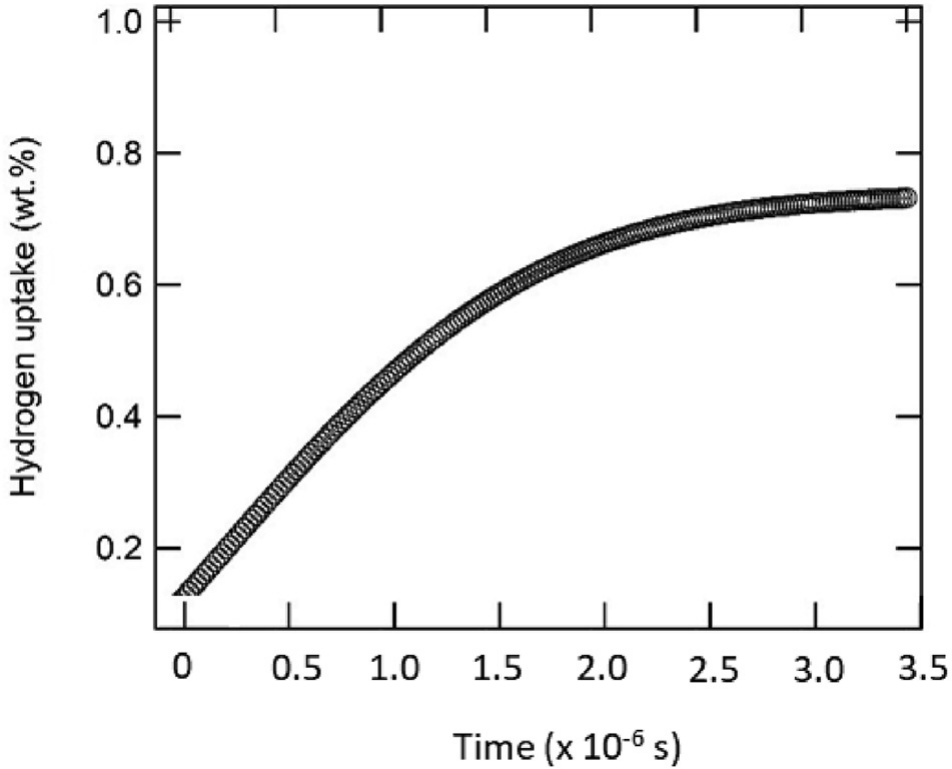
The time evolution of hydrogen uptake of ZSM-5 at 77 K and 0.99 atm.

After obtaining the final state of adsorbed hydrogen in the materials, the calibration to determine the absorbed hydrogen value was conducted by examining the hydrogen uptake as a function of time-step. Figure 3 shows the selected time-varied hydrogen uptake in ZSM-5 at a temperature and pressure of 77 K and 0.99 atm, respectively. It is observed that the hydrogen uptake reaches a point of saturation at 0.77 wt.% after 2.30 × 10^−6^ s. This saturated value is then used as a baseline for further pressure variation while the temperature is kept constant. The simulations for all the different materials show similar features. Therefore, it is possible to construct the isothermal plot between hydrogen uptake and pressure.

**Figure 4:**
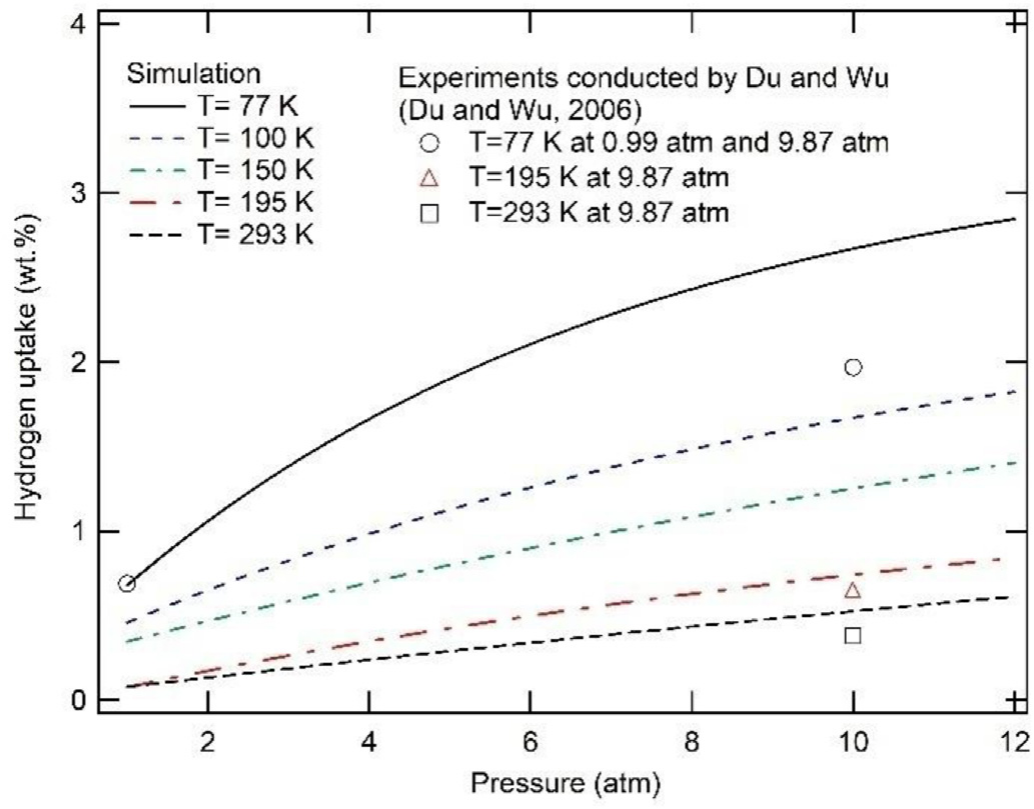
The simulation results of hydrogen uptake isotherms of ZSM-5. The experimental results are plotted based on an experiment conducted by Du and Wu (2006).

The hydrogen uptake in isotherms of ZSM-5 at various temperatures has been depicted by Figure 4, where one can observe that the hydrogen uptake increases almost linearly with pressure, with a change noted at a temperature of 77 K, when the amount of adsorbed hydrogen increases rapidly as the pressure increases in the range between 0.99 and 5.92 atm. Above this pressure, the amount of adsorbed hydrogen in the ZSM-5 continues to increase slowly until it reaches a maximum of about 2.89 wt.% at 12.16 atm. Therefore, it is concluded that the hydrogen adsorption characteristic of ZSM-5 represents the type I isotherm associated with a lower adsorption rate at a higher temperature (Du and Wu, 2006). This characteristic can be explained in thermodynamics, where according to equation ΔG=RTlnk (where ΔG is Gibbs free energy, and *k* is the partition function of the system), the Gibbs energy of the system increases with temperature. The increase in Gibbs free energy provides the system with excess energy, thus encouraging desorption rather than adsorption. Kim Choon Ng and several others developed a universal adsorption isotherm model (Ng et al., 2017) that represents the hydrogen adsorption dependence of site energy and temperature in the following Eq. (2):

(2) $$\theta \left(\varepsilon \right)=\frac{e^{\left(\frac{\Delta \varepsilon -\varepsilon_{c}}{RT}\right)}}{1+e^{\left(\frac{\Delta \varepsilon -\varepsilon_{c}}{RT}\right)}}$$

where θ is the gas adsorption uptake, ε is the site energy, $\varepsilon_{c}=-RT\;\ln\;Kp$_,_ *R* is the gas constant, and *K* is the adsorption constant.

The comparison between this presented study and the available experimental data for ZSM-5 is also presented in Figure 4. In the case of hydrogen adsorption isotherm at 77 K, our simulation obtains a well-matched result with the experimental data, for a low pressure of 0.99 atm. However, at a higher pressure of 9.87 atm, the simulation exhibits a major deviation from the experimental findings. This relative discrepancy is significantly lower for higher temperatures of 195 K and 293 K. At this stage, it is inferred that the deviation at high pressure conditions could be associated with the inaccuracies in the Avogadro equation that describes the hydrogen adsorption. To overcome these inaccuracies, we corrected the gas ideal equation using PV = nRT, generally used in experiments to fit with Eq. (1). However, the modification made with the new equation still produced a notable deviation. Therefore, we ruled out this possibility as a cause. The other available explanation is that at a higher pressure, the adsorption process is governed not only by the interaction of hydrogen atoms with the framework but also by the hydrogen-hydrogen interactions, leading to the enhancement of the quantum effect; and thus resulting in a deviation of hydrogen adsorption capacity (Salazar et al., 2017). Another possible reason for the discrepancy is that the quality of experimental work is highly dependent on the synthesis and operation procedure, whereas in computation, a perfect crystal structure was used to estimate the adsorption isotherm (Li et al., 2012).

**Figure 5:**
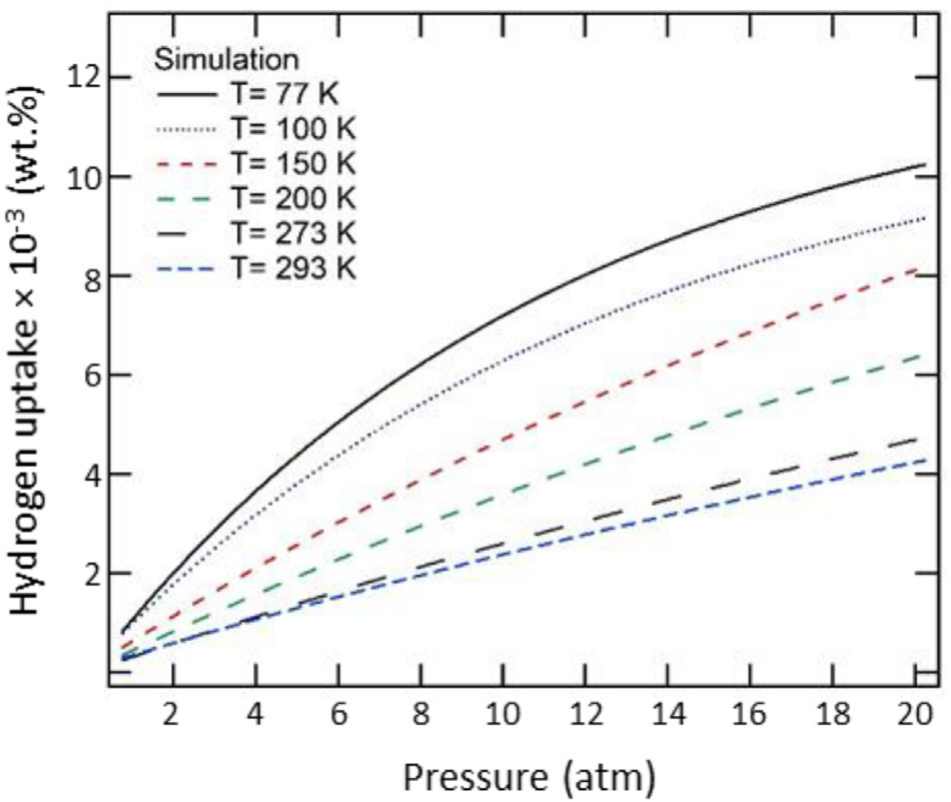
Simulation results of hydrogen adsorption isotherms of GNF.

Figure 5 presents the hydrogen uptake isotherms of GNF at various temperatures. One can be observed that the hydrogen uptake of GNF increases and decreases with pressure and temperature, respectively. At the isothermal temperature from 150 K to 293 K, the amount of hydrogen uptake increases linearly with the pressure. However, at lower temperatures of 100 K and 77K, the hydrogen adsorptions displaying a tendency towards saturation for pressure levels above 20 atm with maximum hydrogen uptake at 20 atm were found to be 0.09wt.% and 0.10 wt.%, respectively. Ahn et al. (1998), who conducted an experiment on hydrogen uptake of GNF, observed that the hydrogen absorbed for 4.44 atm at 77 K, and 18 atm at 300 K, were less than 0.02 and 0.029 wt.%, respectively. Although the exact values were not provided, their reports align with our results where the hydrogen uptake values from our simulation at those conditions are below 0.1 wt.%.

**Figure 6:**
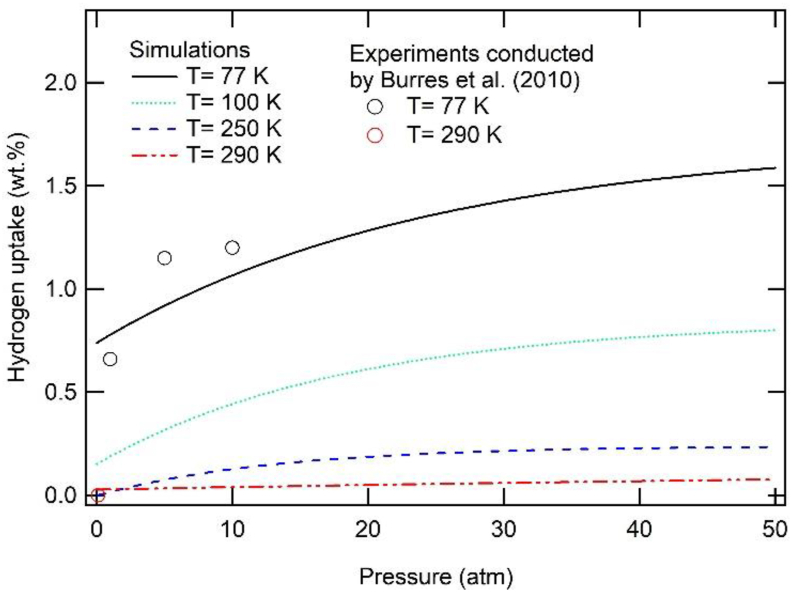
Simulated hydrogen adsorption isotherms at various temperatures for GOF. The experimental results are plotted based on experiments conducted by Burress et al. (2010).

The simulation results of hydrogen uptake for GOF are presented in Figure 6. The structure of GOF in the simulation was drawn from an experiment conducted by Burress et al. (2010) where it had one linker every 32 graphene atoms (GOF-32). The simulation results indicate a decline in the hydrogen uptake value with an increase in temperature. The pressure dependence of hydrogen uptake is observed for isotherm temperatures of 77 K and 100 K, where the notable increase in hydrogen adsorption is observed during an increase in pressure of up to 20 atm. Above 20 atm, the hydrogen adsorption is prone to saturate. On the other hand, at isotherm temperatures of 250 K and 290 K, the increase in pressure does not affect hydrogen uptake. Our results remain in positive alignment with the experimental results reported by Burress et al. (2010), as observed in Figure 6.

**Figure 7:**
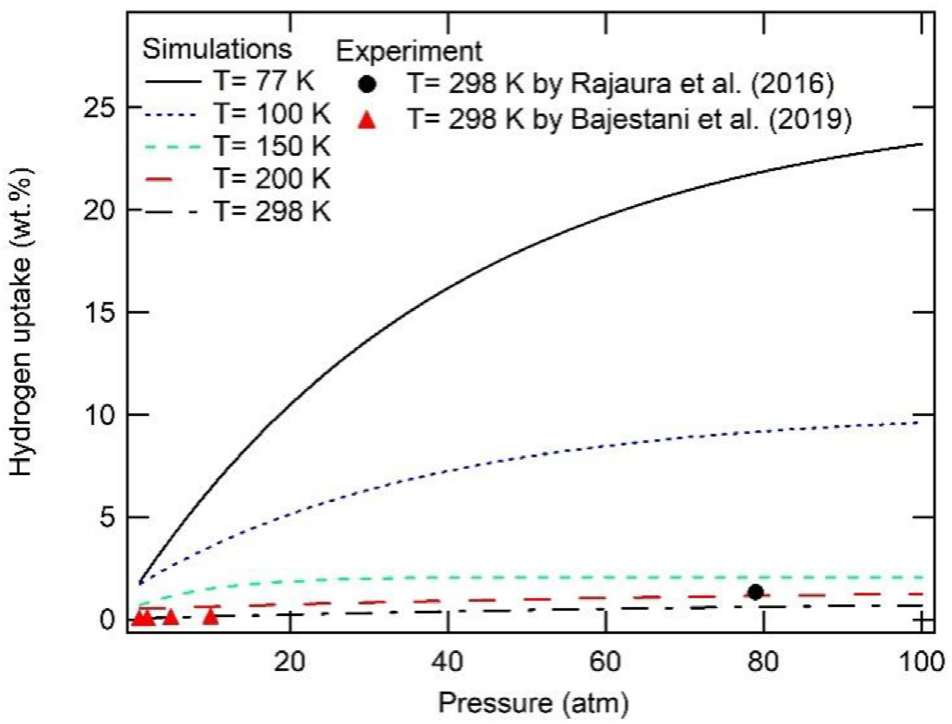
The simulation results of hydrogen uptake isotherms for rGO at various temperatures. The experimental results are plotted based on experiments conducted by Rajaura et al. (2016) and Bajestani et al. (2016).

Figure 7 shows the simulation results of hydrogen adsorption isotherms for rGO, performed at various temperatures. The simulation results of rGO also show a pressure dependence of hydrogen uptake for isotherm temperatures of 77 K and 100 K. These simulation results were then compared to the available experimental data from Rajaura et al. (2016) and Bajestani et al. (2016). At an isotherm temperature of 298 K, this simulation proves to be congruent with the findings from the experiment at low and high pressures, where the value of hydrogen uptakes nearly does not change with the pressure.

From the simulation results, the lowest hydrogen uptakes were observed for GNF. This is due to the lower specific surface area of GNF relative to other carbon materials (Meng and Park, 2010). All the materials exhibit the same characteristics when the hydrogen uptake decreases with the increase in temperature at low and high pressure regions. It is inferred that as the temperature increases, the more kinetic energy was applied to the adsorbents by the system. As a result, the adsorbed materials become unstable, thus decreasing their adsorption energy and leading to a lower chance of adsorption. The change in kinetic energy was confirmed in our simulation and in other reports (Zolfaghari et al., 2007; Strobel et al., 2006; Schlapbach and Zuttel, 2011). In addition, the hydrogen uptake is generally prone to increase proportionally to the amount of pressure; this was inferred from the higher influence of specific surface area and pores on the adsorbents at high pressure compared to those at low pressure (Maulana Kusdhany and Lyth, 2021; Sethia and Sayari, 2016). Overall, from the comparisons between our simulation results and the experiments conducted previously, it is evident that the LAMMPS-based MD offers a reliable estimate of hydrogen molecules adsorption in ZSM-5 and carbon materials, having a prospective potential to predict the adsorption behaviors of other molecules on other complex materials. The reliability of MD simulation in predicting the adsorption affinity of small molecules on carbon nanotubes was also reported by Comer et al. (2015) using CHARMMS, a force field in LAMMPS.

## 4 Conclusion

We have successfully computed the hydrogen adsorption isotherms at various temperatures for ZSM-5, GNF, GOF, and rGO using a simple and low-cost LAMMPS-based MD. From the simulation, it can be concluded that hydrogen uptake value decreases with the increase in temperature for all samples. The simulation results positively correspond with the experiments in all cases at isotherm temperatures of above 77K, proving the reliability of the LAMMPS. At a low isotherm temperature of 77 K, the simulation is congruent with the experiment data to some degree, except at a higher pressure region. We believe our results will promote further computational studies on hydrogen adsorption behavior in other materials using LAMMPS-based MD.

## Declarations

### Author contribution statement

Jaka Fajar Fatriansyah, Donanta Dhaneswara, Iping Suhariadi, Muhammad Ihsan Widyantoro, Billy Adhitya Ramadhan, Muhammad Zaky Rahmatullah, Rahman Hadi: Conceived and designed the experiments; Performed the experiments; Analyzed and interpreted the data; Contributed reagents, materials, analysis tools or data; Wrote the paper.

### Funding statement

This work was supported by Publikasi Terindeks Internasional (PUTI Q2 2020) Universitas Indonesia Grant (NKB1681/UN2.RST/HKP.05.00/2020).

### Data availability statement

Data included in article/supplementary material/referenced in article.

### Declaration of interests statement

The authors declare no conflict of interest.

### Additional information

No additional information is available for this paper.
